# Early Neurodevelopment and Self-Reported Adolescent Symptoms of Depression and Anxiety in a National Canadian Cohort Study

**DOI:** 10.1371/journal.pone.0056804

**Published:** 2013-02-20

**Authors:** C. Rebecca North, T. Cam Wild, Lonnie Zwaigenbaum, Ian Colman

**Affiliations:** 1 School of Public Health, University of Alberta, Edmonton, Canada; 2 Department of Pediatrics, University of Alberta, Edmonton, Canada; 3 Department of Epidemiology and Community Medicine, University of Ottawa, Ottawa, Canada; University of Iowa Hospitals & Clinics, United States of America

## Abstract

**Objective:**

Little is known about the mental health outcomes of young children who experience developmental delay. The objective of this study was to assess whether delay in attaining developmental milestones was related to depressive and anxious symptoms in adolescence.

**Method:**

The sample included 3508 Canadian children who participated in a nationally representative prospective cohort study. The person most knowledgeable about the child reported on attainment of developmental milestones spanning several developmental domains at ages 2–3. The children were followed into adolescence and self-reported depressive and anxious symptoms were used from adolescents ages 12–13. An overall assessment of developmental milestones as well as a supplementary analysis of specific categories of developmental milestones was conducted.

**Results:**

Cohort members who displayed delayed developmental milestones in early childhood were more likely to experience higher levels of depressive and anxious symptoms as adolescents. However, there was no interaction between delayed developmental milestones and stressful life events. In the supplementary analysis, two developmental domains (self-care and speech/communication) were associated with higher levels of depressive and anxious symptoms in adolescence.

**Conclusion:**

Delay in attainment of early developmental milestones is significantly associated with adolescent depressive and anxious symptoms.

## Introduction

Depression is the leading cause of disability worldwide and the seventh leading contributor to the burden of disease [1]. Despite its high prevalence, the causes of depression and the relationships between causal factors remain unclear. Depression has been associated with cognitive deficits [2]–[6], although it is still uncertain whether these are premorbid traits or are a manifestation of the depressive state. Studies which have investigated baseline deficits in memory [2], [3], executive function [2], [4] and attention [2], [5], [6] in individuals with a family history of depression have been equivocal as to whether such deficits precede the onset of depression. However, higher childhood cognitive ability is associated with fewer symptoms of anxiety and depression in adult women [7]. Additionally, lower childhood IQ is associated with more persistent adult depression and higher psychiatric comorbidity [8]. It has been suggested that reductions in childhood IQ may be a marker for neurological differences which increase vulnerability to some mental disorders [8]. Developmental delay is associated with other forms of developmental psychopathology (e.g., autism, ADHD) [9] which have been associated with depression [10], [11]. Studies thus far have only examined cognition in school-aged children; it is unclear whether cognitive deficits in younger children may influence risk for depression.

A small number of studies have examined the association between delay in attaining early developmental milestones and future depressive symptoms. Later attainment of motor developmental milestones is associated with childhood affective disturbance [12]. Similarly, a longitudinal study investigating the trajectory of depressive and anxious symptoms across the life course found that delays in first standing and first walking were significantly associated with depressive and anxious symptoms [13]. In a community sample, later attainment of nocturnal bladder control was significantly related to emotional problems for Chinese adolescents [14] and increased risk of adolescent suicidal behavior [15]. Early developmental milestones are indicative of current cognitive development [16] and are related to subsequent childhood and adult cognitive function [17].

Reaction to psychosocial stress has been suggested as a factor that may influence the association between lower childhood IQ and depression [8]. Individuals with lower childhood IQs may not cope as well with stress, resulting in increased vulnerability to depression after stressful events [8]. To the best of our knowledge, this hypothesis has not been studied in relation to delayed developmental milestones.

### Aim of the Study

The aim of the current study was to investigate the hypotheses that delay in attaining early developmental milestones (at ages 2–3) is associated with depression in adolescence (at ages 12–13), and that exposure to stressful life events exacerbates the effects of delayed milestones on depressive and anxious symptoms. This was achieved and will be explored in the discussion section.

## Materials and Methods

### Source of data

The study used data from the National Longitudinal Survey of Children and Youth (NLSCY), a longitudinal survey conducted by Statistics Canada since 1994/1995 [18]. The sampling and methodology of the NLSCY have previously been described in further detail [19]. The survey began with a sample of 16,903 Canadian children who have been followed prospectively by Statistics Canada every two years. Data were available up to cycle 7 (2006/07) of the survey.

### Sample

The sample consisted of children (unweighted *N* = 3508) who were 2–3 years old in 1994/1995 (*N* = 1500) or 1996/1997 (*N* = 2008) of the NLSCY and responded to self-report measures of depressive and anxious symptoms in 2004/2005 or 2006/2007 (respectively) of the survey, i.e., when they were ages 12–13. The sample included 1737 females and 1771 males.

The follow-up rate for the NLSCY from 1994/1995 to 2006/2007 was 65% [19]. Children who were delayed in achieving their developmental milestones were not significantly more likely to have not been a respondent in 2006/2007 than those who were not delayed. Additionally, weights provided by Statistics Canada were used in the analysis to ensure that the final sample was representative of the Canadian population.

### Developmental Milestones

The Motor and Social Development Scale (MSD scale) assesses motor, social and cognitive development of young children (0–47 months) [20]. The respondent for the MSD scale was the person most knowledgeable (PMK) about the child, who was most often the mother (i.e., 91.3% in 1994/1995 [21] and 91.5% in 1996/1997 [22]). The MSD scale was developed from scales that are commonly used to assess child development (the Bayley, Gesell and Denver scales) and has high reliability and validity [20]. Examples of items include “Has he ever counted out loud up to 10?”, “Has she ever walked up stairs by herself without holding onto a rail?” and “Does he know his own age and sex?” The total score for each child on the 15 items on the Motor and Social Development Scale was the total number of ‘yes’ (whether or not the child had achieved the milestone) answers for the 15 questions. This score was age standardized by age in months [23]. Delay was operationalized by classifying respondents with MSD raw scale scores that were one standard deviation below the mean [24] or greater as ‘delayed’; all other respondents were classified as ‘not delayed’.

### Anxiety and Depression Symptom Scale

Youths aged 12–13 years self-reported common symptoms of anxiety and depression [25]. The seven items included “I am unhappy or sad”, “I am not as happy as other people my age”, “I am too fearful or nervous”, “I worry a lot”, “I cry a lot”, “I am nervous, highstrung or tense”, and “I have trouble enjoying myself”, with three response options: “never or not true”, “sometimes or somewhat true”, or “often or very true”. The scores for each of the seven items were totaled to give the overall score for the scale. These items were derived from the Child Behavior Checklist by investigators for the Ontario Child Health Study on the basis that they appeared to operationalize DSM-III criteria for emotional disorder [25]. The Child Behavior Checklist is a widely used scale that assesses common symptoms of behavioral and emotional problems [26]. Subjects were then divided into four groups of scores by level of symptoms of depression and anxiety experienced (the score categories were divided to be approximately: 0–50%, no symptoms; 51–75%, mild symptoms; 76–90%, moderate symptoms; and 91–100%, severe symptoms), in order to capture variation in severity of symptoms. This grouping of depressive symptoms is the same procedure that has been previously used by other researchers [13], [27], [28]. The weighted mean (SD) overall scale score was 0.95 (0.82) for the no symptoms group, 3.48 (0.50) for mild symptoms, 5.84 (0.79) for moderate symptoms, and 9.33 (1.51) for the severe symptoms group.

### Stressful Life Events

In the longitudinal NLSCY cohort, the PMK for children 4 to 15 years of age was asked “In the past 2 years, has this child experienced any events or situations that caused him/her a great amount of worry or unhappiness?” If the answer to this question was yes, then the PMK was asked to indicate the cause(s) from a list of potential stressful life events (i.e., Death of Parents, Death in Family, Divorce/Separation of Parents, Move, Death of Pet, Stay in Hospital, Stay in Foster Home, Other Separation from Parents, Illness/Injury of Child, Illness/Injury of Family Member, Abuse/Fear of Abuse, Change in Household Members, Alcoholism or Mental Health Disorder in Family, Other Traumatic Events). If children had experienced one or more event since the time of the last interview cycle, they were classified as having a recent stressful life event. We also examined the effects of stressful life events prior to the past two years (beginning at age 2–3). Both recent and previous stressful life event variables were categorized as none, one, or two or more stressful life events.

### Covariates

Several potential confounders were included in multivariate analyses. Infant girls score significantly higher than infant boys on the MSD scale [20]. Also, females have higher rates of depression than males; this gender gap is established by adolescence [29]. Consequently, gender was controlled for in all multivariate analyses.

Another potential confounder is race [20], [30]. White children score higher on the MSD scale than minority children [20]. Additionally, it has been suggested that culture may shape how an individual experiences and expresses symptoms of depression [30]. Subjects were thus categorized as “White” and “Non-White”.

Family type is related to children's MSD scores. Children who are from families including step parents, or father only families had lower scores, while those from two-parent and mother-only families had average scores and those from adopted families had higher than average scores [31]. Parental divorce is associated with increased symptoms of distress in adolescence [32]. Family type was operationalized as a three category variable: single-parent families, step-parent families and families including both biological parents.

Socioeconomic status has been considered a potential confounder in studies investigating developmental milestones [14]. Also, depressive symptoms are more common among lower socioeconomic status individuals [33]. Socioeconomic status was operationalized as the ratio of household income, controlling to the ‘low-income cut off’ score. This measure considers an individual's income relative to the community in which they live as well as the size of their family [34]. The NLSCY divided the measure into six levels. Due to sample size restrictions, the current study collapsed the measure into three levels.

Gestational age was considered as a confounder in the multivariate analysis. Babies who are born preterm have slower development [31]. Within the range of full term gestation, a lower gestational age is associated with depressive symptoms (independent of birth weight) [35]. Gestational age was used as a continuous variable.

Children who were 2–3 years old in both 1994/1995 and 1996/1997 were combined into a single sample. To account for this, the cycle from which the data came was controlled for in the adjusted models.

### Secondary Analysis

In a secondary analysis, developmental milestones were separated into four categories: self-care, communication, gross motor and fine motor. Self-care, communication and gross motor milestones were continuous variables. However, the NLSCY only contained one question on fine motor milestones in 2–3 year old children, so this was operationalized as a dichotomous variable. Scores were inversed so that a higher score represented fewer milestones having been attained. As these categories were not age-standardized, age in months was considered as an additional covariate.

### Statistical Analysis

Ordinal logistic regression was used to explore the relationship between developmental delay and depressive and anxious symptoms at ages 12–13. Three models were tested including the fully adjusted analysis (all potential covariates), the partially adjusted analysis (gender, socioeconomic status and cycle) and the crude analysis. Previous authors have suggested that there may be sex-specific associations in the relationship between early cognitive ability and adolescent depression. Given this finding we also conducted sex-stratified analyses for all models [7]. The proportional odds assumption was tested for all models and not violated. Bootstrap weights provided by Statistics Canada were included in the analysis to accommodate for the survey design and to ensure that the sample was representative of the Canadian population. STATA 10 was used to conduct the analysis.

### Ethics

The study was approved by the Health Research Ethics Board of the University of Alberta.

## Results

Descriptive statistics for the sample are presented in Table 1. The distribution of anxious/depressed symptoms are presented in Table 2.

**Table 1:** Descriptive information for sample.

Characteristic	Weighted Percentage/Weighted Mean (SD)
Age in Months	35.99 (6.73)
Cycle (Two)	48.39%
MSD Score (Continuous; Overall)	100.31 (14.36)
MSD Score (Continuous; 2 Year Olds)	100.10 (14.19)
MSD Score (Continuous; 3 Year Olds)	100.51 (14.52)
Gender (male)	51.02%
SES (mid)	7.38%
SES (high)	74.69%
Race (White)	88.51%
Family Type (intact)	82.25%
Family Type (not intact)	6.44%
Gestational Age (Days)	272.93 (14.54)
Emotional Disorder/Anxiety Score	3.39 (2.73)
Previous SLE (1)	35.11%
Previous SLE (2 or more)	26.75%
Recent SLE (1)	29.22%
Recent SLE (2 or more)	4.20%

**Table 2:** Weighted distribution of anxious/depressed symptoms.

	No Symptoms	Mild	Moderate	Severe
Delay	39.61%	24.69%	28.59%	7.11%
No Delay	44.49%	24.62%	23.07%	7.82%

Children aged 2–3 years with delay in reaching developmental milestones had increased odds of higher anxious and depressive symptoms at ages 12–13 in both the fully adjusted and partially adjusted models (Table 3). In the stratified analysis for females, delay was not a significant predictor in any model. In the stratified analysis for males, delay was a significant predictor in all models (Table 3).

**Table 3:** Association between delayed milestone attainment at age 2–3 and symptoms of depression and anxiety in adolescence.

	Males	Females	Overall
Crude	*1.39 (1.00–1.93)	1.49 (0.90–2.49)	1.20 (0.91–1.57)
Adjusted^1^	*1.42 (1.02–1.98)	1.42 (0.86–2.35)	*1.39 (1.06–1.82)
Adjusted^2^	*1.43 (1.03–1.98)	1.46 (0.87–2.48)	*1.41 (1.08–1.85)

1 Adjusted for gender, SES and cycle (with the exception of gender in analyses stratified by gender).

2 Adjusted for gender, race, SES, family type, gestational age and cycle (with the exception of gender in analyses stratified by gender).

* p<0.05.

No significant interaction effects were found between delayed milestone attainment and both recent or previous stressful life events on the likelihood of higher anxious and depressive symptoms.

### Secondary Analysis

For children aged 2–3 years, decreased attainment of both self-care and communication milestones was associated with an increased odds of higher depressive and anxious symptoms in both the partially and fully adjusted models (Table 4).

**Table 4:** Association between categories of developmental milestones and depressive symptoms in adolescence.

	Males	Females	Overall
**Self Care**			
Crude	***1.23 (1.09–1.38)	1.05 (0.87–1.27)	1.08 (0.98–1.19)
Adjusted^1^	**1.26 (1.09–1.46)	1.07 (0.88–1.30)	**1.18 (1.06–1.32)
Adjusted^2^	**1.27 (1.10–1.47)	1.09 (0.89–1.33)	**1.20 (1.07–1.33)
**Communication**			
Crude	***1.21 (1.10–1.34)	*1.15 (1.01–1.31)	***1.14 (1.06–1.23)
Adjusted^1^	***1.22 (1.10–1.37)	**1.21 (1.05–1.41)	***1.23 (1.12–1.34)
Adjusted^2^	***1.23 (1.10–1.37)	*1.22 (1.04–1.42)	***1.24 (1.13–1.35)
**Gross Motor**			
Crude	1.10 (0.97–1.24)	1.06 (0.91–1.23)	1.06 (0.96–1.18)
Adjusted^1^	1.07 (0.92–1.25)	1.12 (0.94–1.32)	1.08 (0.97–1.22)
Adjusted^2^	1.07 (0.92–1.25)	1.13 (0.95–1.34)	1.10 (0.98–1.23)
**Fine Motor**			
Crude	1.18 (0.87–1.60)	0.96 (0.74–1.25)	0.94 (0.78–1.14)
Adjusted^1^	1.12 (0.79–1.58)	0.99 (0.75–1.32)	1.04 (0.84–1.29)
Adjusted^2^	1.06 (0.76–1.50)	0.99 (0.74–1.32)	1.03 (0.83–1.28)

1 Adjusted for gender, age in months, SES and cycle (with the exception of gender in analyses stratified by gender).

2 Adjusted for gender, age in months, race, SES, family type, gestational age and cycle (with the exception of gender in analyses stratified by gender).

* p<0.05.

** p<0.01.

*** p<0.001.

In the stratified analysis of children aged 2–3, decreased attainment of communication milestones was significantly related to an increased odds of higher depressive and anxious symptoms in both males and females in all models. Decreased self-care milestones were associated with an increased odds of higher depressive and anxious symptoms in males but not in females (Table 4).

Gross and fine motor milestones were not associated with depressive and anxious symptoms.

## Discussion

In this study of 3508 Canadian adolescents followed from birth, there was a significant association between delay in attaining early developmental milestones and increased odds of higher depressive and anxious symptoms at ages 12–13. This is consistent with a study which found that delay in first standing and walking were associated with depressive symptoms across the life course [13]. Previous studies have linked lower cognitive ability among school-aged children to increased [7] and more persistent [8] depressive symptoms, suggesting that cognitive deficits precede the onset of depression. The results of the current study suggest that these cognitive deficits may be present at 2–3 years of age.

There are a number of factors that could affect the relationship between delays in achieving developmental milestones and adolescent depressive and anxious symptoms. Relative to their typically developing peers, children with developmental delays are more likely to have behavioral problems. The development of behavioral problems in this population is affected by child characteristics including temperament and self-regulatory ability as well as by family factors such as family stresses and parent-child interactions [36]. Developmental delay is associated with forms of developmental psychopathology (e.g., autism, ADHD) [9] that have been associated with depression [10], [11]. This could represent a possible mediator of the relationship between delays and anxious and depressive symptoms.

No significant interactions were found between either recent or previous stressful life events and delay in attaining developmental milestones. These results do not support a stress-diathesis model for early delays and depression. For recent stressful life events, we used parent report measures of stressful life events over the past two years or since the time of the last interview. This has three important limitations. First, in adolescence the median duration of a depressive episode is nine months [37]. Therefore, by the time of assessment of depressive symptoms, the potential increase in depressive symptoms after a stressful life event may have already passed. Second, parents may not be aware about potential school and peer-related stressful life events in a child's life. Hence, there may have been some misclassification in determining whether children had experienced a stressful life event. Additionally, one of the main limitations of our study relates to the wording of the question concerning stressful life events. This is discussed in detail below.

In the secondary analyses, decreased attainment of self-care and communication milestones was significantly associated with increased odds of experiencing higher depressive and anxious symptoms in adolescence. These results suggest that the relationship between delay in attaining developmental milestones and depressive and anxious symptoms in adolescence is not specific to one developmental domain. However, the relationship may be more important for milestones relating to cognition than those related to motor domains.

In the current study, delayed attainment of motor milestones was not significantly related to depressive and anxious symptoms in adolescence. This contrasts with findings in previous research. One longitudinal study found that delays in first standing and first walking were associated with depressive and anxious symptoms [13]. This study asked mothers of two year olds to recall the age in months when the child first stood and walked without assistance. In contrast, the motor milestones used in the present study, such as pedaling a tricycle at least ten feet and walking up stairs without a rail, are attained at an older age. Hence, the relationship between the attainment of motor milestones and adolescent symptoms of anxiety and depression may not be as important in later stages of child development (ages 2–3).

Decreased attainment of communication milestones was associated with an increased odds of higher depressive and anxious symptoms in adolescence. This is consistent with findings that children with expressive language delay are more likely to experience social-emotional problems [38]. Similarly, there is comorbidity between child communication disorders and child psychiatric disorder [39]. Additionally, adolescent speech problems have been linked to adult psychiatric disorder [40]. Our findings appear to be consistent with prior studies.

Additionally, when males and females were stratified, there was a significant association between decreased attainment of self-care milestones and higher depressive and anxious symptoms in males, but not females. A study of parenting conducted across 110 societies demonstrated that parents exerted pressure on girls to be nurturing and responsible whereas more pressure was put on boys to be self-reliant and to strive for achievement [41]. It may be that achievement of independence from caregivers is both culturally and, in turn, psychologically more important for boys. This could underlie the gender difference in the relationship between these early milestones and adolescent mental health. This could represent an environmental modulator of cognitive vulnerability to depression.

There are four main limitations to the study. First, the measures used in this study do not include a clinical diagnosis of depression, but rather, rely on self-reported symptoms of depression. However, recent findings suggest that subthreshold depressive disorders are common and negatively affect health [42]. By using the self-report measure of depressive symptoms, this study may have the advantage of capturing some of these subthreshold depressive disorders. The second limitation of this study is that early developmental assessments are limited to a parent-reported questionnaire on developmental milestones rather than validated developmental screening tools or developmental assessments by health professionals. However, early developmental milestones are indicative of current cognitive development [16] and are related to subsequent childhood and adult cognitive function [17]. It is possible that parents may have the potential to forget or miss milestone attainment and then misreport. However, such misreporting is unlikely to be related to later emotional stability in the child, suggesting non-differential misclassification and consequent conservative estimates presented in this paper. The third limitation of this study relates to the measure of stressful life events. The wording of the question is problematic in that it asks the PMK to report on any events or situations in the past two years that caused the child a great amount of worry or unhappiness. This may have made the PMK for children who had emotional problems more likely to report that there was an event that caused the child worry or unhappiness particularly for the “Other Traumatic Events” category. However, the majority of the categories (e.g., Death of Parent) are independent stressors and would be expected to be reported evenly for children with and without emotional problems. Previous researchers investigating the nature of the causal relationship between stressful life events and depression have classified stressful life events based on whether they could have resulted from an individual's behaviour or were likely to be independent events [43]. We would expect that the majority of categories of stressful life events listed in the current study to be independent stressful life events. There would be no reason to expect those with delay attaining developmental milestones to report stressful life events differently than those without delay. Therefore, if bias were present as a result of the wording of this question, we would expect it to be towards the null. This limitation may have contributed to our non-significant findings. The fourth limitation of this study is that outcomes were measured in 12–13 year olds. A recent study suggests that the relationship between cognitive ability in childhood and depressive symptoms in adolescence is dependent on age and/or pubertal stage [44]. Specifically, the study found that child IQ at age 8 was inversely associated with depressive symptoms at age 11. However, at age 13–14 the association reversed and a higher IQ score at age 8 was associated with higher risk of symptoms of depression [44]. The current study examined 12–13 year olds and thus these results may be affected by subjects with differing ages and pubertal stages. In the stratified analysis, developmental delay was a significant predictor of depression for males, but not females. This may be due to the fact that males undergo puberty later than females.

These limitations are offset by notable strengths, including the prospective design, and a large sample size representative of the Canadian population.

The results imply that early cognitive development is related to adolescent mental health. These findings suggest that cognitive deficits precede the onset of depression and are not solely a state characteristic of depression, but are also likely to be a pre-morbid trait of some individuals who develop depression.
